# Screening of molluscicidal strain against *Oncomelania hupensis* from the rhizosphere of medicinal plant Phytolacca acinosa Roxb

**DOI:** 10.4103/0973-1296.66928

**Published:** 2010

**Authors:** Danzhao Guo, Jun Chen, Xiangping Du, Bangxing Han

**Affiliations:** 1 *College of Pharmacy, Jiangsu University, Zhenjiang, Jiangsu P. R. China*; 2 *College of Life Science and Technology, Henan Institute of Science and Technology, Henan, P. R. China*

**Keywords:** ITS sequence, molluscicidal activity, *Oncomelania hupensis*, *Phytolacca acinosa* Roxb, rhizosphere, strain SL-30

## Abstract

The research aimed to screen and exploit molluscicidal microorganisms against *Oncomelania hupensis*, from the rhizosphere of medicinal plant, *Phytolacca acinosa* Roxb., and one strain named as SL-30 was obtained with excellent molluscicidal activity. The freeze-dried powder of exocellular broth (EXB) of SL-30 could kill 100% of snails at a concentration of 48 mg/l for a submerged period of 24 h, with stabile molluscicidal activity at a temperature lower than 60°C; furthermore, it could be gradually degraded after exposure to illumination for 15 days. The freeze-dried powder of SL-30’s EXB was safe to fresh fish and shrimp, even at a concentration beyond LC_90_ of 24-h exposure period. The glycogen and total protein content of soft tissues of snails decreased after treating with SL-30’s EXB, and glycogen content’s decreasing rate had a linear relationship with molluscicidal activity. Finally, phylogenetic analysis based on ITS sequence showed that strain SL-30 had a higher similarity to *Aspergillus fumigatus* with bootstrap value 98%; accordingly, it was identified as a species of *Aspergillus*.

## INTRODUCTION

Schistosomiasis remains a parasitic disease prevailing in many parts of the developing countries.[1] In China, schistosomiasis caused by *Schistosoma japonicum* is epidemic in the southeast, especially in the areas of Yangtze River valley.[2] Snail (*Oncomelania hupensis*) is the only intermediate host of *S. japonicum*.[3] Therefore, one of the effective preventive methods against schistosomiasis is the control of snails to break the life cycle of the parasite. Niclosamide is the only synthetic molluscicide compound recommended by World Health Organization (WHO).[4] However, there have been problems with the application of niclosamide in a large scale, such as high cost of synthesis, potential resistance of snail to the action of drug, acute and potential toxicity to non-target organisms,[5] and so on. Consequently, it is urgent to screen a safe, cheap, effective and readily available alternative biomolluscicide.

Since the 1950s, a few molluscicidal microbial strains have been studied, but ultimately they were not applied in practice owing to instability of the molluscicidal substances at high temperature or in visible light, difficulty in transportation of the cells, and so on.[6] Nevertheless, compared with other organisms, microorganisms have the diversity of species type and metabolic pathway (or metabolic product), short growth period, and the yield ability is inducible.[7] Based on this, continuous search for microbial molluscicidal agents with a rational and effective method is one of the most promising categories in biomolluscicides. The rhizosphere is the region of soil surrounding plant roots, which is under the influence of the root,[8–10] and in which there are more types and quantities of microorganisms, compared with the non-rhizosphere region.[11] Furthermore, there are abundant special exudates, including medicinal secondary natural products, in the rhizosphere of medicinal plants,[12] which forms a natural enriched medium for microorganisms. As a result, from the rhizosphere of medicinal plants, it is possible to obtain potential isolates producing the bioactive substance which may be same, similar or relative to the secondary metabolites of the host plant. In fact, some microbial strains producing biological factors relative to the corresponding medicinal host, have been isolated from the rhizosphere.[13,14] But to our knowledge, microbial molluscicide against *O. hupensis*, from the rhizosphere region has not been reported. Based on the reported molluscicidal properties of *Phytolacca acinosa* R*oxb*., which is one of the traditional Chinese medicines,[15,16] this study focused on screening molluscicidal microbial strain from the rhizosphere of P. acinosa Roxb., and finally one strain exhibiting high molluscicidal activity was obtained and was named as SL-30. At the same time, the stability and acute toxicity and preliminary physiological and biochemical effect of SL-30’s exocellular broth (EXB) on *O. hupensis* were examined, and finally, taxonomic status was determined with phylogenetic analysis. The following experimental work provided a foundation for purifying, optimizing and studying the active substance contained in SL-30’s EXB, and for the further practical application.

## MATERIALS AND METHODS

### Soil samples and strains

Soil samples were taken from the rhizosphere of *P. acinosa* R*oxb*. in Anhui and Jiangsu Province. Plant roots were gently shaken to remove the adhering soil, then samples were collected from the regions at about 2 mm distance to the surface of root system and were encased in sterilized kraft paper envelopes, and treated in 1 week as quickly as possible.

Strains were isolated from the soil samples according to the routine method.[17] Approximately 10 g of soil was scattered in 100 ml sterile water in flasks and shaken on rotary shaker at 100 rpm for 30 min to dislodge cells from the soil particles, thus forming a diluent 10^–1^, and the diluents were diluted 10 fold in sterile water until a diluent 10^–6^ is formed. Then, 0.1 ml of the diluent 10–2, 10^–3^ and 10^–4^ was inoculated on the surface of Martin’s medium by coating, 0.1 ml of diluent 10^–3^, 10^–4^ and 10^–5^ on Gause’s Synthetic Agar, and 0.1 ml of diluent 10^–4^, 10^–5^ and 10^–6^ on Nutrient Agar. Then they were incubated at 27°C for 3–7 days. The representative colonies which appeared on the medium were transferred to a new agar plate with an inoculating loop, and then well-isolated single colonies were examined with light microscope (OLYMPUS, Tokyo, Japan) as the final pure stains.

### Preparation of exocellular broth

The potential isolates were inoculated into Martin’s liquid medium flasks at 27°C for 60 h for bacteria, 108 h for fungi and 168 h for actinomycetes. Then, the broth was centrifuged at 5000 rpm for 10 min to remove biomass impurities, and the supernatants (namely EXB), which were adjusted to pH 7.0 by adding 0.1 M HCl solution or 1 M NaOH solution, were diluted with dechlorinated tap water to 20, 10 and 5%, respectively, for molluscicidal activity bioassay.

### Source of animals

Snails (*O. hupensis*) were collected from the beach of the Yangtze River near Zhenjiang and acclimatized to the laboratory conditions at room temperature (25 ± 1°C) for 2–3 days. Then, the healthy adult snails with seven to eight spirals were selected for the test.

Zebra fish (*Brachydanio rerio*) and peacock fish (*Poecilia rericulata*) were bought from the market with a mean fresh weight of 0.38 g and mean length of 3 cm. Shrimp (*Macrobrachium nippoensis*), with a mean wet weight of 3.0 g and mean length of 3 cm, were collected from the pond in the campus of Jiangsu University. Zebra fish, peacock fish and shrimp were acclimatized to the test conditions in continuous aeration domestication for 1 week, and then healthy and lively individuals were selected for the test.

### Molluscicidal activity bioassay

Molluscicidal activity against *O. hupensis* was determined with the immersion method as per WHO recommendations.[18] At room temperature (25 ± 1°C), three bags of live snails as one group (30 snails/bag) were submerged into beakers containing 900 ml of the test solutions and were kept soaked for 24, 48 and 72 h, respectively. At the same time, 1 mg/l of niclosamide aqueous was adopted as the positive control, and dechlorinated tap water and blank medium as two negative control groups. Each test was set in triplicate on three different days. Then, the test snails were removed from the bags, washed with dechlorinated tap water and then placed into dechlorinated tap water to be observed for 3 days. The snails climbing upward the wall of beakers were judged to be alive, and the snails that remained in the bottom of beakers were further examined to check mortality by mechanical prodding. The ratio of dead snails to total tested snails was expressed as mortality (%).

#### Lethal concentration (LC_50_ and LC_90_) values of freeze-dried powder of SL-30’s EXB

The SL-30’s EXB was freeze-dried into powder. The powder was made into concentrations of 8, 16, 24, 32, 40 and 48 mg/l with dechlorinated tap water for molluscicidal test. The data were handled with SPSS 13.0 for LC_50_ and LC_90_ values.

#### Detection of stability of freeze-dried powder of SL-30’s EXB

The freeze-dried powder of SL-30’s EXB was prepared with dechlorinated tap water at the concentration of LC_90_ for 24-h exposure period, and divided into three equal parts treated in the following three ways, respectively: (1) heated at different temperatures (40°C, 60°C, 80°C and 100°C) for 30 min, with room temperature (25 ± 1°C) as control; (2) heated for different periods (30, 60, 90, 120, 150 and 180 min) at the same temperature (80°C), with room temperature (25 ± 1°C) as control; and (3) placed in an incubator with constant light intensity (1200 lux) and temperature (25 ± 1°C) for different periods (1, 3, 5, 7, 10, 15, 20 and 30 days).

The stability of the molluscicidal active substances contained in the EXB was evaluated according to the molluscicidal activity of the solutions mentioned above.

#### Acute toxicity of freeze-dried powder of SL-30’s EXB to freshwater fish and shrimp

Toxicity of freeze-dried powder of SL-30’s EXB to fish and shrimp was assessed through a 96-h semi-static exposure test with 24-h renewal.[19] The test vessels were plastic buckets containing 10 l of test concentration which was renewed every 24 h. Temperature during the test period was set at 25 ± 1°C. Each group of 20 fish or shrimp was exposed to the concentration of 8, 16, 24, 32, 40 and 48 mg/l, respectively. Dechlorinated tap water was adopted as negative control. The experiment was set in triplicate. The animals were not fed during the experiment, and the dead animals were removed immediately. The mortality (%) of each group was calculated and recorded at 24, 48, 72 and 96 h, respectively.

#### Glycogen and total protein assay

The freeze-dried powder of SL-30’s EXB was prepared at concentrations of 8, 12, 16 and 20 mg/l, respectively, with dechlorinated tap water, which were less than the concentration of LC_50_ in the case of submerged period 24 h. Then, 60 snails were submerged in beakers containing the test diluent for 24 h. At the same time, the negative group test was performed with the dechlorinated tap water. Each test was set in triplicate. After being immersed for 24 h, the living snails were picked out from the beaker, and the soft tissue of each snail was obtained after removing the shell and weighed before and after vacuum drying for 12 h at 40°C. Then, ratios of dry weight (DW)/fresh weight (FW) of the snails were calculated, and the dried soft tissue was further ground into fine powder for glycogen and total protein assay.

About 10 mg of the soft tissue powder was weighed and encased in the test tube. Then, 2 ml solution of 30% KOH was added to the tube and incubated in a boiling water bath for 20 min. After incubating, the test tube was cooled to room temperature. Finally, 10 ml ethanol was added and the precipitate (namely glycogen) was got by centrifuging at 3000 rpm for 10 min. Glycogen content was analyzed by anthrone-colorimetric method.[20]

Approximately 100 mg of the soft tissue powder was used, and the nitrogen content was determined by Kjeldahl nitrogen detection method.[21] The total protein content was calculated as: total protein % = 6.25 × N%.

### Statistical analysis

The test data were statistically analyzed by SPSS13.0. The effect of SL-30’s EXB on *O. hupensis* was expressed by LC_50_ and LC_90_ values and their 95% confidence limit (95% cl).

### Phylogenetic analysis

Strain SL-30 was cultured in Martin’s liquid medium for 48 h. The fresh mycelia were obtained by centrifuging at 5000 rpm for 10 min; then the mycelia were ground in liquid nitrogen into fine powder for genomic DNA extraction.[22] The genomic DNA was extracted with UNIQ-10 DNA extraction kit (Shanghai Sangon, China) according to the instruction. The rDNA-ITS region was amplified by polymerase chain reaction (PCR) using the general primers ITS1 (5′-TCCGTAGGTGAACCTGCGG-3′) and ITS4 (5′-TCCTCCGCTTATTGATATGC-3′). PCR was carried out as follows. The total volume of the reaction mixture was 50 μl and contained 10 pmol DNA template, 1 μl each primer (10 μmol/l), 1 μl NTP mix (10 mmol/l each), 5 μl 10× PCR buffer, 0.25 μl Taq polymerase (5 u/μl), and water. PCR was performed in a thermal cycler (My cycler 1.065, bio-rad, USA) as follows: 98°C for 5 min, followed by 35 cycles of 95°C for 35 s, 55°C for 35 s, 72°C for 40 s, and with a final extension at 72°C for 8 min. PCR products were electrophoresed in 1% low-melting temperature agarose gel, visualized with ethidium bromide, and excised separately. The separated PCR products were further purified with UNIQ-10 PCR Purification Kit (Shanghai Sangon, China). The sequence of the purified rDNA-ITS was analyzed by the Shanghai Sangon Biological Engineering Technology and Services Co. Ltd. (Shanghai, China).

The rDNA-ITS sequences were submitted to Genbank. The reference rDNA-ITS sequence data were obtained from the GenBank nucleotide sequence database. All the sequences were aligned with the multialignment program CLUSTAL W.[23] The aligned sequences were corrected manually, focusing on gap positions. DNA sequence data were analyzed to provide pairwise percentage sequence divergence. A phylogenetic tree was produced according to the neighbor joining with software of MEGA version 3.1. Clade stability was assessed with 1000 bootstrap replications.

## RESULTS

### Molluscicidal activity of the strains isolated from the rhizosphere

**Figure 1:**
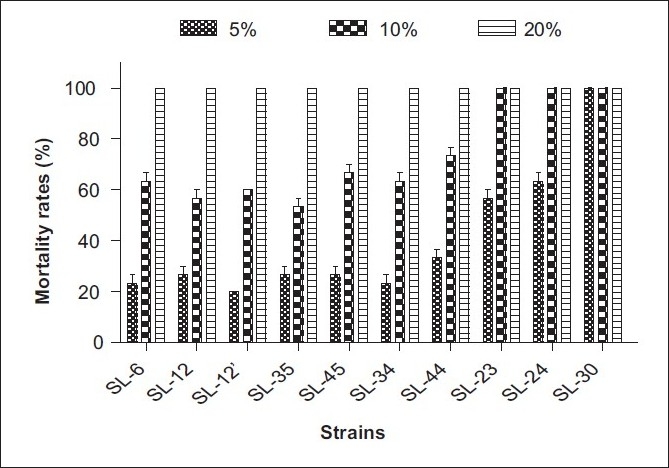
Molluscicidal activity against *O. hupensis* of partial strains from the rhizosphere of *P. acinosa Roxb*

A total of 116 pure strains were isolated from the rhizosphere of *P. acinosa* R*oxb*., and their molluscicidal activity against snails within a 48-h exposure period is indicated as follows. When diluted to 20%, the EXB of 10 strains showed excellence with a mortality of 100%. But when further diluted to 10%, only three strains remained with the mortality of 100%. Finally, one strain numbered SL-30 was obtained which gave the mortality of 100% when diluted to 5% [Figure 1], and it had higher molluscicidal activity than the previous work reported.[18] Therefore, the following work focused on strain SL-30.

### LC_50_ and LC_90_ values of freeze-dried powder of SL-30’s EXB

The freeze-dried powder of SL-30’s EXB showed good molluscicidal activity, and the LC_50_ and LC_90_ values were 25.5 and 43.5 mg/l, respectively, in the case of exposure time 24 h [Table 1]. But when the exposmure time was increased up to 48 and 72 h, the LC_50_ and LC_90_ values were too low to be detected and calculated.

**Table 1:** Molluscicidal effect of freeze-dried powder of SL-30's EXB

Submerged periods (h)	Molluscicidal activity (mg/l)	
	LC_50_ (95% cl)	LC_90_ (95% cl)
24	25.5 (21.97–34.28)	43.5 (35.16–61.07)
48	NA	NA
72	NA	NA

NA: not available

### Stability of freeze-dried powder of SL-30’s EXB

**Figure 2:**
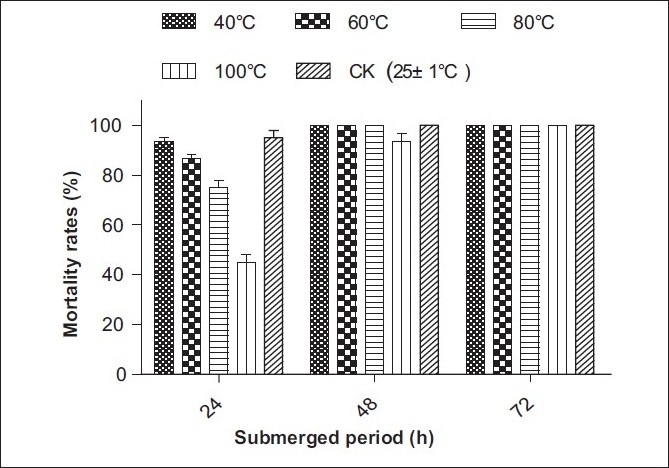
Stability of freeze-dried powder of SL-30's EXB against heating

**Figure 3:**
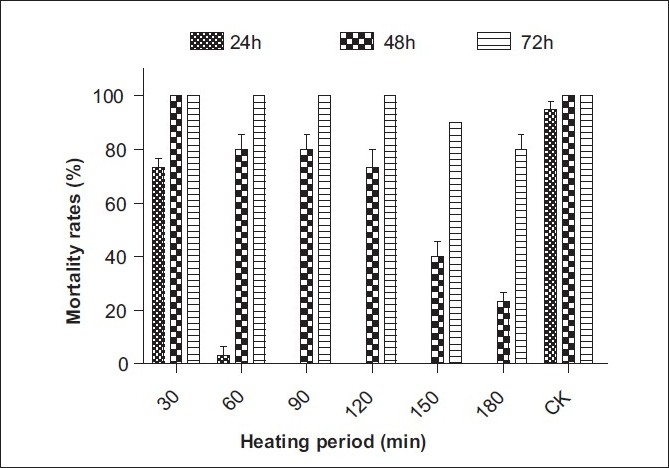
Stability of freeze-dried powder of SL-30's EXB at 80°C

**Figure 4:**
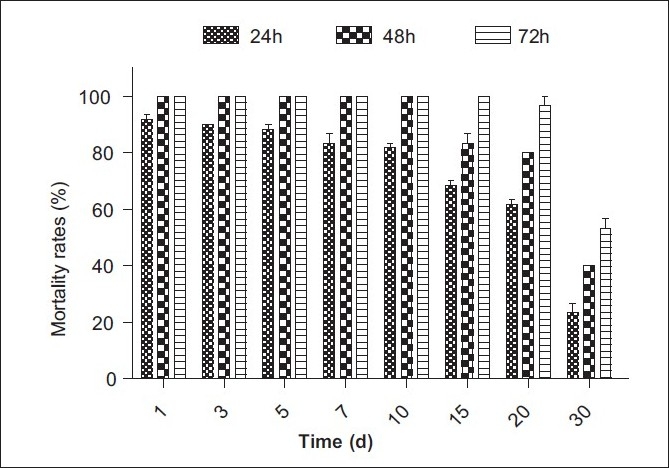
Stability of freeze-dried powder of SL-30's EXB against illumination

The SL-30’s EXB was prepared at a concentration of 43.5 mg/l with dechlorinated tap water (LC_90_ value of 24-h exposure period), and then the stabilities of the molluscicidal active substances were investigated. The results were as follows. After the samples were heated at 60°C, 80°C and 100°C for 30 min, the molluscicidal activity with the submerging period of 24h could be reserved to a different extent (85–45%), but which was stable by treated with 40°C. Meanwhile, the mortality with the submerging period of 48 and 72 h was still 100% except for being heated at 100°C [Figure 2]. After heating at 80°C for 30 min, the molluscicidal activity with the submerging period of 24 h gradually decreased to 75%, then dropped acutely when heated for further longer time [Figure 3]. The molluscicidal activity of 24-h exposure period was stabile within 15 days and then degraded slowly under a light intensity of 1200 lux [Figure 4]. It indicated that the active substance contained in SL-30’s EXB was almost thermostabile at the temperature lower than 60°C and possessed molluscicidal activity within 30 days at room temperature. That is to say, the active substance could have enough time to play its role of controlling snail in the natural environment before it was completely degraded; on the other hand, it was biodegradable and therefore friendly to the environment.

**Figure 5:**
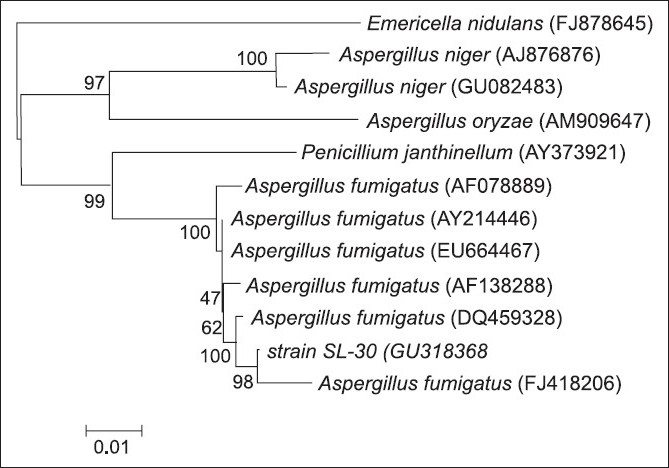
Phylogenetic tree of strain SL-30 based on rDNA-ITS analysis

### Acute toxicity of freeze-dried powder of SL-30’s EXB to freshwater fish and shrimp

Each treatment with the test concentration was safe for peacock fish and shrimp with a mortality of zero [Table 2]. For zebra fish, the EXB was safe within exposure time of 72 h in all test concentrations, and the mortality of 48 mg/l, which was more than the concentration of LC _90_ of 24-h exposure period, was as low as 3.3% when exposure time lasted up to 96 h. It demonstrated that the toxicity of SL-30’s EXB to snails was far strong than to freshwater fish and shrimp in the same concentration.

**Table 2:** Mortality of fish and shrimp treated by freeze-dried powder of SL-30's EXB (*n* = 3)

Animals	Exposure time (h)	Mortality with different dose (mg/l)						
		8	16	24	32	40	48	N
Zebra fish	24	0	0	0	0	0	0	0
	48	0	0	0	0	0	0	0
	72	0	0	0	0	0	0	0
	96	0	0	0	0	0	3.3% ± 0.028	0
Peacock fish	24	0	0	0	0	0	0	0
	48	0	0	0	0	0	0	0
	72	0	0	0	0	0	0	0
	96	0	0	0	0	0	0	0
Shrimp	24	0	0	0	0	0	0	0
	48	0	0	0	0	0	0	0
	72	0	0	0	0	0	0	0
	96	0	0	0	0	0	0	0

N, dechlorinated tap water as negative control

### Glycogen and total protein content of tested snail’s soft tissues

No obvious changes of average DW and FW of soft tissues were observed after the treatments and the ratios of DW/FW were between 18 and 20% [Table 3]. But the glycogen content decreased greatly after treating with SL-30’s EXB, ranging from 18.28 to 53.23%, and the decreasing ratio was parallel to the molluscicidal activity [Table 4] and it suggested that the active substance contained in the EXB influenced the snail possibly by interfering with the energy metabolism. The total protein content also decreased after treatments, ranging from 14.24 to 16.76% [Table 5], but the protein content decreasing ratio was disproportional to the molluscicidal activity.

**Table 3:** Weight of soft tissues of snails after treating with freeze-dried powder of SL-30's EXB (*n* = 3)

Item	Concentration of freeze-dried powder (mg/l)				
	N	8	12	16	20
Average FW (mg/snail)	24.33 ± 1.12	22.46 ± 1.35	21.76 ± 1.28	21.58 ± 1.31	24.23 ± 1.16
Average DW (mg/snail)	4.51 ± 0.30	4.45 ± 0.23	3.94 ± 0.21	4.01 ± 0.12	4.50 ± 0.31
DW/FW (%)	18.54 ± 2.21	19.81 ± 1.67	18.11 ± 2.15	18.58 ± 2.31	18.57 ± 2.12

N, dechlorinated tap water as negative control

**Table 4:** Glycogen Content of soft tissues of snails after treated with freeze-dried powder of SL-30's EXB (*n* = 3)

Item	Concentration of freeze-dried powder (mg/l)				
	N	8	12	16	20
Content of glycogen (mg/g dry powder)	31.13 ± 1.21	25.44 ± 1.01	22.64 ± 1.11	16.98 ± 1.22	14.56 ± 1.15
Decreasing ratio (%)	—	18.28 ± 1.32	27.27 ± 1.77	45.45 ± 1.39	53.23 ± 1.52

N, dechlorinated tap water as negative control

**Table 5:** Total protein content of soft tissues of snails treated by freeze-dried powder of SL-30's EXB (*n* = 3)

Item	Concentration of freeze-dried powder (mg/l)				
	N	8	12	16	20
Content of protein (mg/g dry powder)	43.32 ± 1.01	36.06 ± 2.34	37.15 ± 1.92	36.52 ± 1.88	36.41 ± 2.13
Decreasing ratio (%)	–	16.76 ± 2.41	14.24 ± 2.01	15.70 ± 1.93	15.95 ± 2.25

N, dechlorinated tap water as negative control

### Taxonomic status of strain SL-30

A sequence of rDNA-ITS about 576 bp was obtained by PCR from the genomic DNA, and with the accession number GU318368 assigned by GenBank. With the addition of sequences from GenBank, the phylogenetic tree was constructed by the neighbor joining methods using *Emericella nidulans* (FJ878645) as the outgroup strain to root the tree. Strain SL-30 was clustered with *Aspergillus fumigatus* (FJ418206) in the phylogenetic tree, and the bootstrap value was very high (98%) [Figure 5]. Accordingly, the rDNA-ITS sequence analysis indicated that the sequence of strain SL-30 had a higher similarity to that of *Aspergillus* sp., and the differences were located in ITS1 and ITS2 areas, both belonging to variable region. Until now, applying the strains of *A. fumigatus* to control *O. hupensis* has been not reported.

## DISCUSSION

Here we have reported that a strain named as SL-30 with good molluscicidal activity was isolated from the rhizosphere of *P. acinosa* R*oxb*. In the case of submerged period 24 h, the LC_50_ and LC_90_ values of freeze-dried powder of SL-30’s EXB were 25.5 and 43.5 mg/l, respectively, which showed better molluscicidal activity compared with the ones previously reported.[18] Then, stain SL-30 was identified as a strain of *A. fumigatus* by phylogenetic analysis. To our knowledge, some strains belonging to *A. fumigatus* were reported previously that they could produce bioactive substances such as taxol[24] and ginkgolides;[25] however, this is the first report saying that *A. fumigatus* possesses molluscicidal activity.

The results indicated that exposure to sublethal concentrations of SL-30’s EXB on snails could influence glycometabolism and protein synthesis. After treating with SL-30’s EXB, glycogen content decreased greatly, ranging from 18.28 to 53.23%, and the decreasing ratio was parallel to the molluscicidal activity. Similarly, the total protein content also decreased after treatments, but the decreasing ratio was disproportional to the molluscicidal activity. The influential factor on glycometabolism of snails was complicated, which mainly focused on the following three aspects. Firstly, it induces partial liver cell necrosis by affecting hepatic function, leading to a direct impact on glycogen synthesis. Secondly, it activates or passivates some enzymes, thus promoting glycogen decomposition and inhibiting glycogen synthesis, resulting in a decrease of glycogen content. Finally, it affects the digestive tract function and causes reduction of cibation and glucose uptake, thus inhibiting glycogen synthesis.[20,26] In addition, the results showed a reduction of total protein content to some extent, but it was less significant than the amount of glycogen decrease. Accordingly, the lethal effect of the active substance on snails might be more related to energy metabolism of cells. Therefore, further experiments are necessary to be carried out to reveal the molluscicidal mechanism.

The preliminary test showed that the EXB of SL-30 was safe to fresh fish and shrimp, and could be gradually degraded in the analogic natural conditions. Accordingly, strain SL-30 is highly promising to be applied in practice. Besides further exploiting the active substance contained in the EXB as a kind of microbial molluscicide through industrial fermentation, the strain SL-30 also can be expected to be applied in the natural breeding position of *O. hupensis*. That is to say, in the breeding place of *O. hupensis*, if strain SL-30 can be colonized in the soil on which growing molluscicidal plants, a molluscicidal ecosystem will be constructed, in which there are synergistic molluscicidal effects between plants and microorganisms. In fact, many studies have indicated that microorganisms could be colonized in the environment and played important roles in respective fields.[27–29] Meanwhile, molluscicidal ecosystem combined with the role of microorganisms was suggested as a novel idea to control *O. hupensis*.[6] Thus, once the stable molluscicidal ecosystem forms, it is promising to provide long-term, continuous and environmentally safe molluscicidal effect. This would help to eliminate or inhibit *O. hupensis* to a large extent.
